# Supplementary material for: Molecular Properties of Human Guanylate Cyclase-Activating Protein 3 (GCAP3) and Its Possible Association with Retinitis Pigmentosa
Source: Int J Mol Sci. 2022 Mar 17;23(6):3240. doi: 10.3390/ijms23063240 (PMC8948881; doi:10.3390/ijms23063240)
Supplement: Supplementary file 1 [file ijms-23-03240-s001.zip › SUPPLEMENTARY_FILES/SI_Avesani_Bielefeld_etal_ijms2022_FINAL.pdf]

International Journal of *Molecular Sciences*

MDPI

Supplementary Materials

Molecular Properties of Human Guanylate Cyclase-Activating Protein 3 (GCAP3) and Its Possible Association with Retinitis Pigmentosa

Anna Avesani, Laura Bielefeld, Nicole Weisschuh, Valerio Marino, Pascale Mazzola, Katarina Stingl, Tobias B. Haack, Karl-Wilhelm Koch and Daniele Dell'Orco

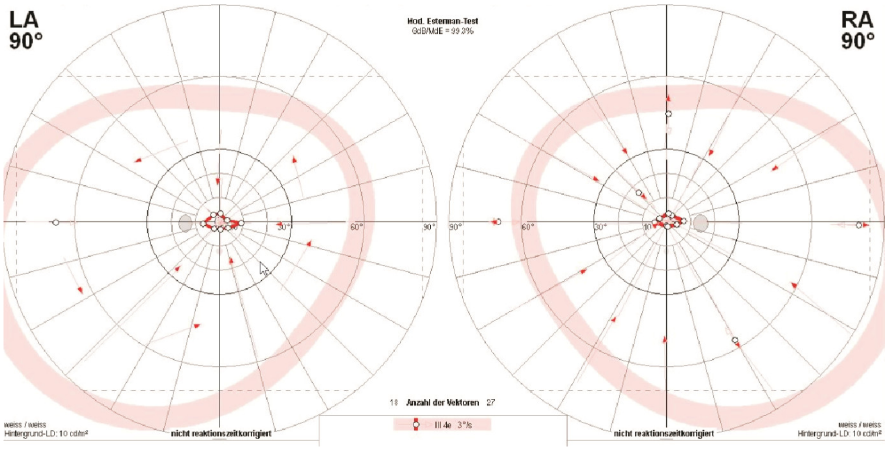

Figure S1. Visual field analysis of the left and right eye of the study patient. The visual field is restricted to 5 degrees.

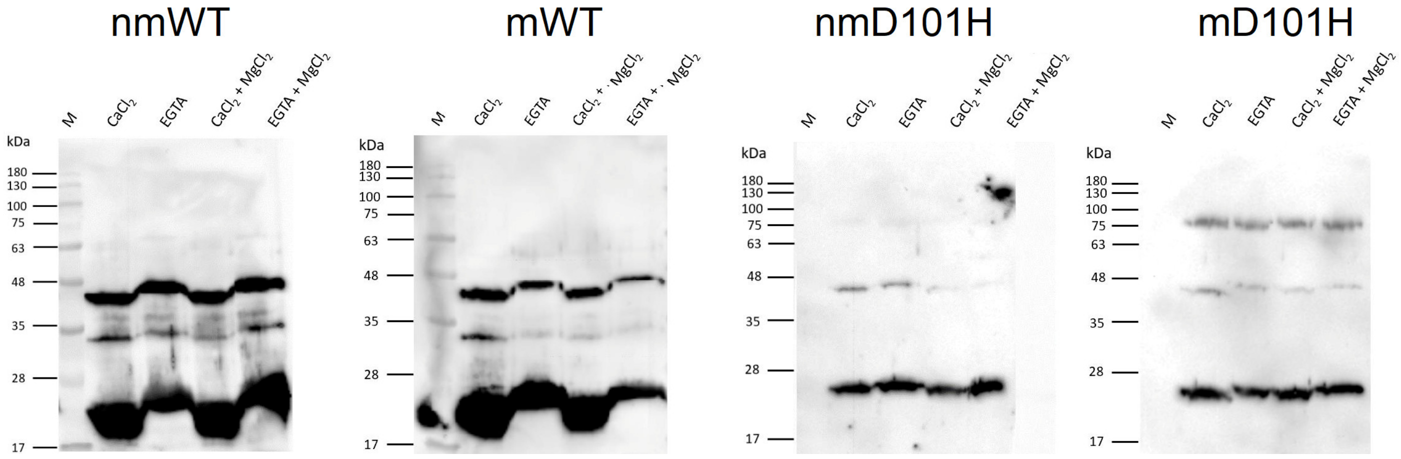

Figure S2. Gel-shift assay combined with western blot of GCAP3 variants. 12% SDS-PAGE of 5 µg nm/m WT and D101H GCAP3 upon incubation with 1 mM Ca^{2+}, 1 mM EGTA, 1 mM Ca^{2+} + 1 mM Mg^{2+} or 1 mM EGTA + 1 mM Mg^{2+}.

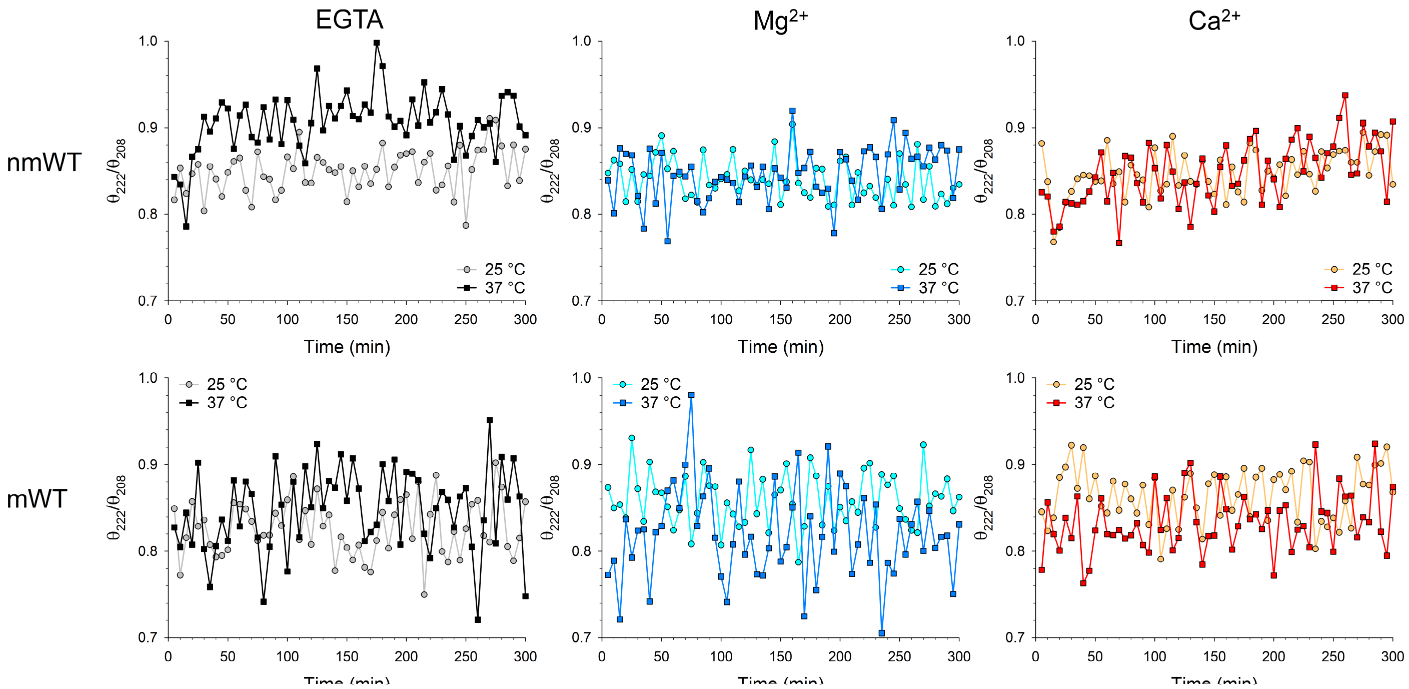

Figure S3. Time evolution over 5 h of the ratio between the ellipticity at 222 and 208 nm ($\theta_{222}/\theta_{208}$) of 8 μM non-myristoylated (upper panels) and myristoylated WT GCAP3 in the presence of 300 μM EGTA (left), 300 μM EGTA + 1 mM Mg^{2+} (center), 300 μM Ca^{2+} (right). Data were collected in the same conditions at 37 °C (black, blue and red, respectively) and at 25 °C (grey, cyan and orange, respectively).

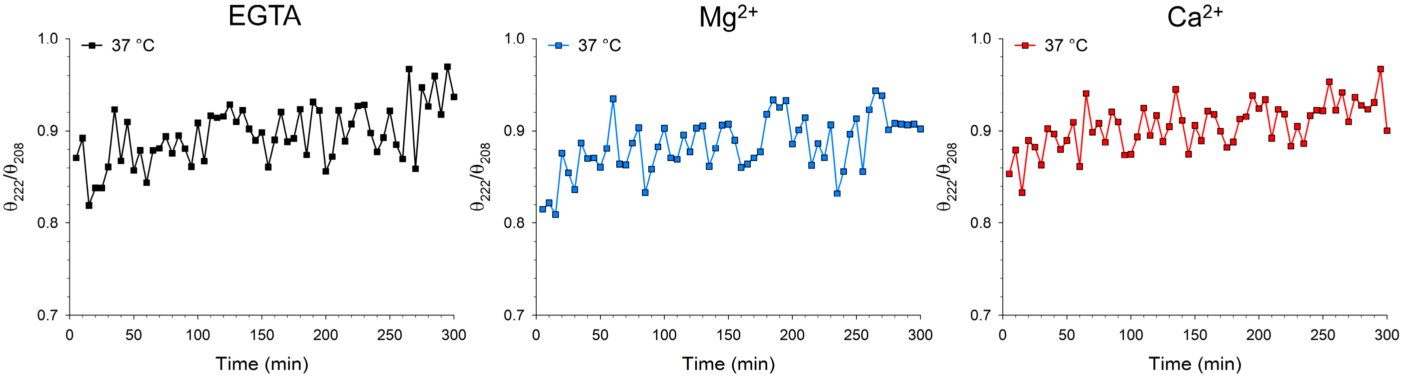

Figure S4. Time evolution over 5 h of the ratio between the ellipticity at 222 and 208 nm ($\theta_{222}/\theta_{208}$) of 8 μM nm D101H GCAP3 in the presence of 300 μM EGTA (left, black), 300 μM EGTA + 1 mM Mg^{2+} (center, blue), 300 μM Ca^{2+} (right, red).

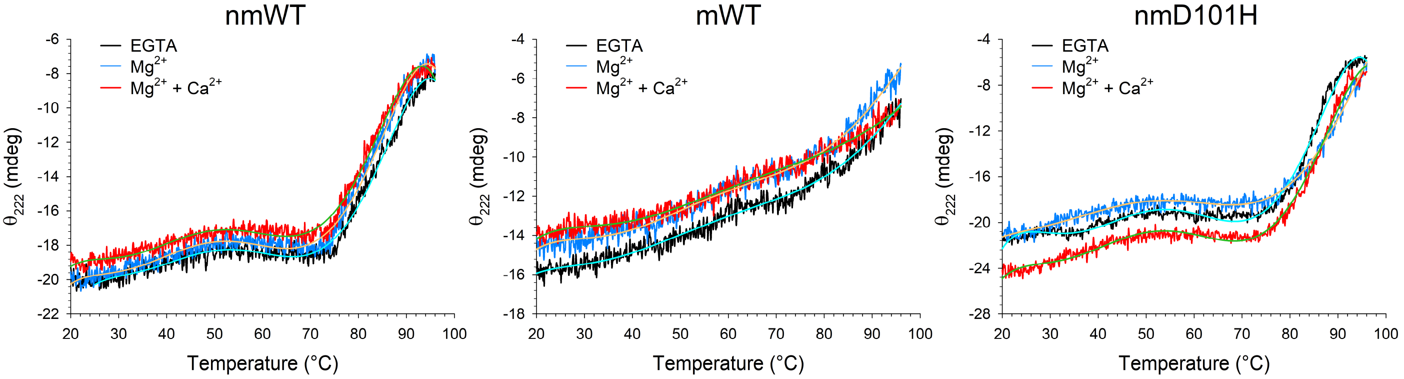

Figure S5. Thermal denaturation profiles of 8 μM non-myristoylated (left) and myristoylated (center) WT and non-myristoylated D101H (right) GCAP3 in the presence of 300 μM EGTA (black), 300 μM EGTA + 1 mM Mg^{2+} (blue) or 300 μM free Ca^{2+} (red). Data were fitted to a polynomial function (in cyan, yellow and green, respectively) which allowed the estimation of the T_m reported in Table 1, obtained by analyzing the first and second derivative as described in the methods section.

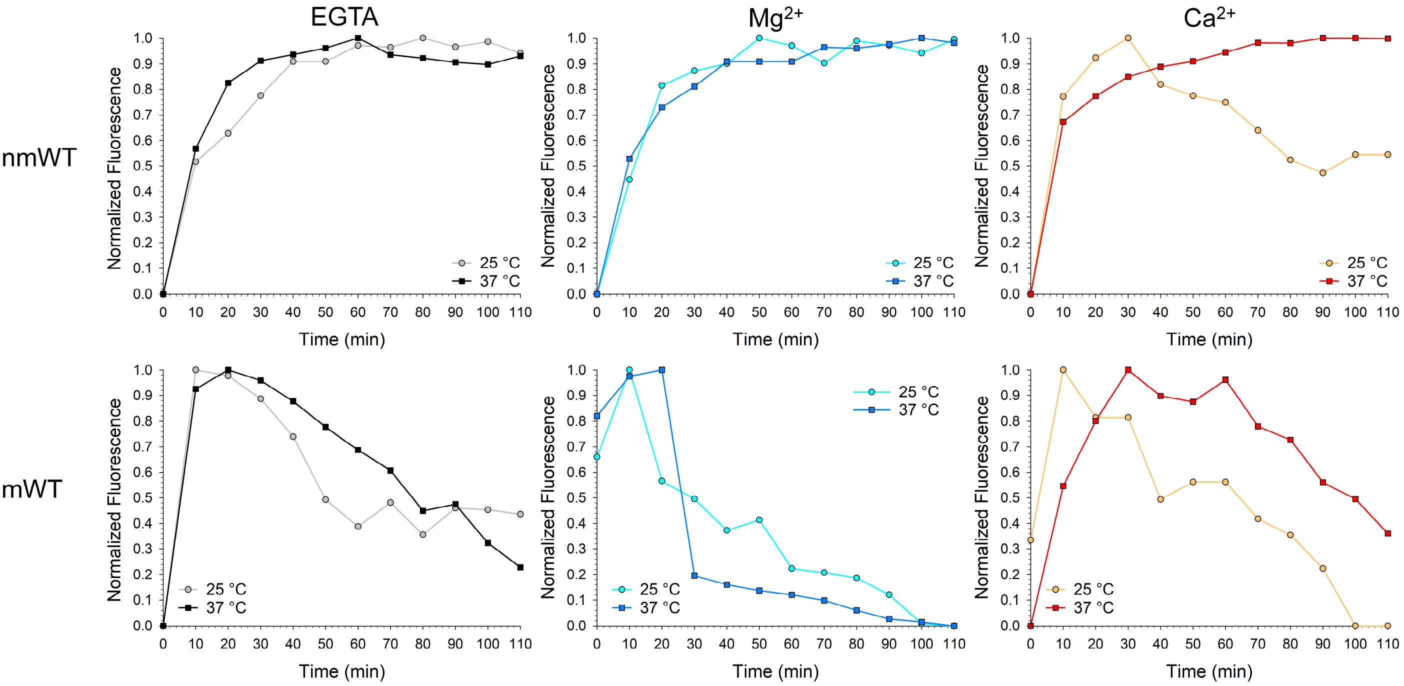

Figure S6. Time evolution over 110 min of the normalized ANS fluorescence of 2 μM non-myristoylated (upper panels) and myristoylated WT GCAP3 in the presence of 500 μM EGTA (left), 500 μM EGTA + 1 mM Mg^{2+} (center), 1 mM Mg^{2+} + 500 μM free Ca^{2+} (right). Data were collected in the same conditions at 37 °C (black, blue and red, respectively) and at 25 °C (grey, cyan and orange, respectively).

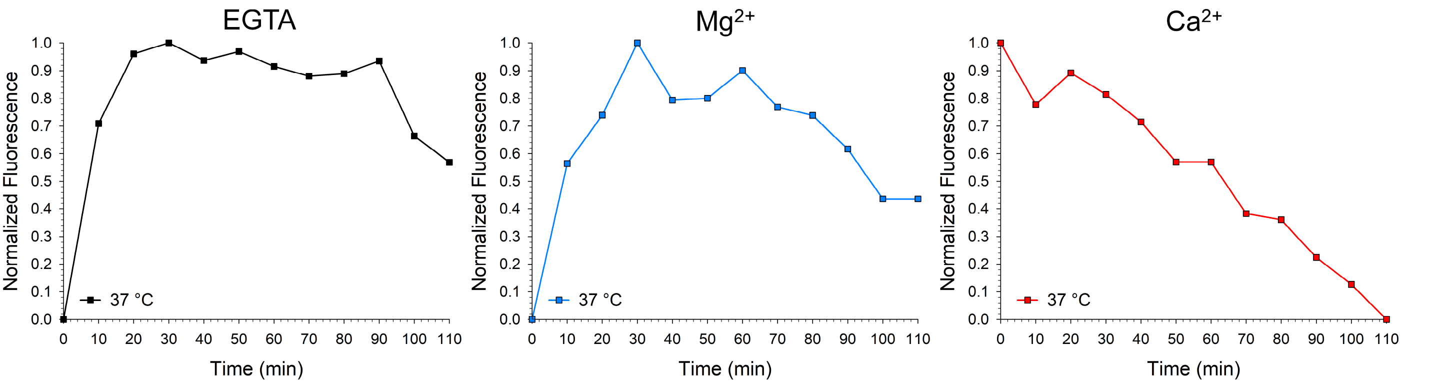

Figure S7. Time evolution over 110 min of the normalized ANS fluorescence of 2 μM nmD101H GCAP3 in the presence of 500 μM EGTA (left, black), 500 μM EGTA + 1 mM Mg^{2+} (center, blue), 1 mM Mg^{2+} + 500 μM free Ca^{2+} (right, red).

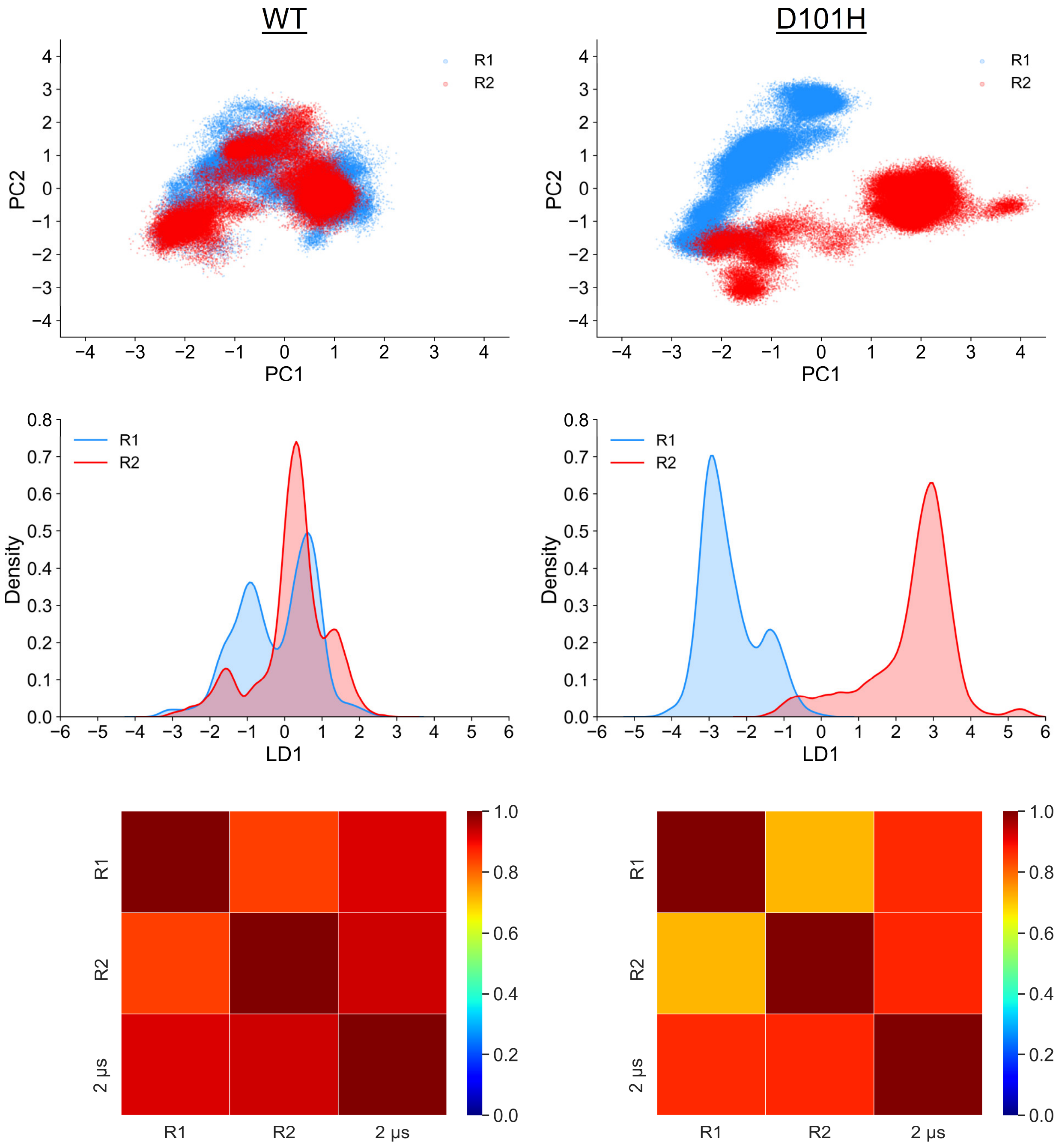

Figure S8. Assessment of the reproducibility of MD simulations (2 replicas, 1 μs each) of WT (left column) and D101H (right column) GCAP3 by PCA. 2D-projection of the trajectories along the first two principal components (top row) and Linear Discriminant Analysis of the projections (middle row), data from R1 and R2 are shown in blue and red, respectively. RMSIP of the first 20 PC extracted from the 2 replicas and the concatenated trajectory (2 μs) against one another. RMSIP values are displayed in a color scale from blue (0, no overlapping of eigenvectors) to red (1, identical eigenvectors). Overall, the overlap of the 2D-projections, the LDA density functions and RMSIP values indicate that the trajectories for both variants are reproducible and consistent, moreover they suggest that nmWT may explore a smaller number of conformations (RMSIP > 0.83) and therefore is inherently more reproducible than nmD101H, which may evolve towards different energetic basins, yet still driven by the same collective motions, as shown by the overlap of the essential subspaces (RMSIP > 0.718).

Video S1. Dissociation of the Ca^{2+}-ion from EF3 monitored by MD simulations. Protein structure is shown as cyan cartoons, Ca^{2+}-coordinating residues are shown as sticks with N atoms in blue and O atoms in red, Ca^{2+}-ion is represented as a green sphere.
